# Fetal Bowel Dilatation due to Intestinal Neuronal Dysplasia: A Rarity

**Published:** 2016-04-24

**Authors:** Arzu Akdag, Karar Orkun Anadut, Omer Yalcin, Mete Kaya

**Affiliations:** 1 Division of Neonataology, Bursa Sevket Yilmaz Training and Research Hospital, Bursa, Turkey; 2 Department of Pediatrics, Bursa Sevket Yilmaz Training and Research Hospital, Bursa, Turkey; 3 Department of Pathology, Bursa Sevket Yilmaz Training and Research Hospital, Bursa, Turkey; 4 Department of Pediatric Surgery, Bursa Sevket Yilmaz Training and Research Hospital, Bursa, Turkey

**Keywords:** Intestinal obstruction, Intestinal neuronal dysplasia, Newborn

## Abstract

Intestinal neuronal dysplasia (IND) type B is characterized by malformation of parasympathetic plexus and manifests at more than 6 month of age with progressive severe constipation. We report a case of IND type B presented with bowel dilatation on antenatal scan and neonatal intestinal obstruction which is unusual with this type of IND.

## CASE REPORT

A full-term male neonate weighing 2290gm born to a 21-year old woman (gravida 1, para 1) by spontaneous vaginal delivery, was admitted to with suspicion of intestinal atresia. Antenatal scan, done at 26-week of gestation, showed marked dilatation of bowel loops (16mm). After 4 weeks, follow-up ultrasonography suggested an increase in the diameter and the number of loops (20mm). On physical examination, mild tachypnea and abdominal distension were observed. Abdominal x-ray showed small bowel dilatation. Because of suspicion of intestinal atresia, an exploratory laparotomy was performed. At surgery approximately 50 cm of bowel distal to the ligament of Treitz was dilated, and an obstruction was found at the end of this segment. The area of obstruction consisted of a dilated meconium-filled proximal bowel segment, an abrupt transition zone, and a distal bowel that was small in caliber (Fig. 1). Jejunal enterotomy was performed and bowel was decompressed. The 10-cm long distal jejunal segment including transitional zone was resected. Stoma was not made and end-to-end anastomosis was done. The disparity of lumen between the proximal and distal segments was five fold. Distal bowel wash-outs with gastrograffin were started on postoperative day five. Genetic study for cystic fibrosis was negative. Histopathological examination of the resected bowel segment established the diagnosis of IND (Fig. 2) on the basis of hyperganglionosis with giant ganglia, ectopic ganglion cells in the lamina propria, and positive immune-histochemical staining for the S-100 protein. Unfortunately, enzyme histochemistry for acetylcholinesterase (ACH) was not performed owing to lack of fresh tissue. A final diagnosis of type B IND was made. Oral feeding was started on day 10 after the operation, and was gradually increased. Patient was discharged on postnatal day 27. He is doing fine on 6-month follow-up.

**Figure:**
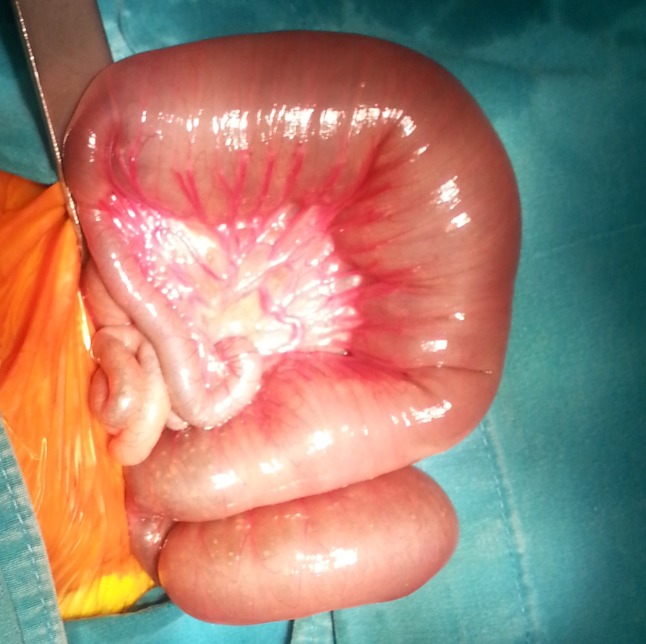
Figure 1:Dilated bowel approximately 50 cm of distal from the ligament of Treitz dilated, and an obstruction at the end of this segment.

**Figure:**
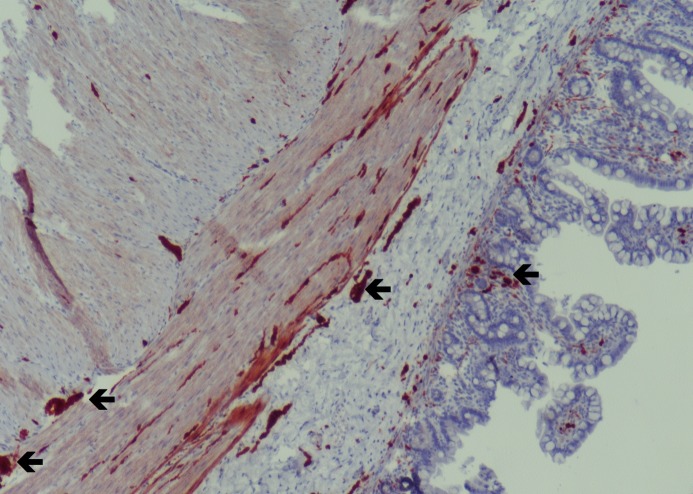
Figure 2:Hyperganglionosis with giant ganglia, ectopic ganglion cells in the lamina propria by immunohistochemical staining for the S-100 protein.

## DISCUSSION

IND was first described in 1971 by Meyer-Ruge as a hyperplastic malformation of the enteric plexus.[1] IND is classified into two clinical and histologically subtypes as types A or B.[2] Type A of IND is congenital hypogenesis or agenesis of the sympathetic innervation, which occur in less than 5% of all IND cases. Type B of IND is characterized by hyperplasia of the parasympathetic plexus and accounts for 95% of all IND cases. IND type A manifests with episodes of abdominal distension, bowel obstruction and diarrhea with blood in stool in neonatal period. Type B IND presents with chronic constipation with or without abdominal distension after 6 months of age.[3] In our case, the presence of antenatal dilated bowel loops was suggestive of fetal bowel obstruction; moreover patient presented with neonatal inetstinal obstruction. Antenatal identification reduces the perinatal morbidity and mortality by early resuscitation and suitable surgical interventions except in rare situations.[4] Our differential diagnosis on the basis of history and abdominal radiograph was that of intestinal atresia.

Typical histological features of IND type B are hyperganglionosis, giant ganglia, and ectopic ganglion cells and increased ACH enzyme activity in the lamina propria and around submucosal blood vessels.[5] Our patient had similar findings however ACH enzyme was not performed. To summarize, IND type B presenting with fetal bowel dilations and neonatal intestinal obstruction is a rare occurrence. IND must be kept in differential diagnosis of fetal bowel dilatation on antenatal scan.

## Footnotes

**Source of Support:** Nil

**Conflict of Interest:** None declared

